# Smart Polymer Composite Deck Monitoring Using Distributed High Definition and Bragg Grating Fiber Optic Sensing

**DOI:** 10.3390/s22114089

**Published:** 2022-05-27

**Authors:** Stephen Young, Dayakar Penumadu, Andrew D. Patchen, George Laggis, Joey Michaud, Abram Bradley, Ryan Davis, John Unser, Matthew Davis

**Affiliations:** 1 Tickle College of Engineering, The University of Tennessee, Knoxville, TN 37996, USA; dpenumad@utk.edu (D.P.); apatche2@vols.utk.edu (A.D.P.); emichau1@vols.utk.edu (J.M.); abradl11@vols.utk.edu (A.B.); rdavi128@vols.utk.edu (R.D.); 2 Walker Engineering and Project Management LLC, Knoxville, TN 37928, USA; gello@baldriver.com; 3 Composite Applications Group, McDonald, TN 37353, USA; junser@iacmi.org; 4 The Institute for Advanced Composites Manufacturing Innovation, Knoxville, TN 37932, USA; 5 LUNA Innovations, Inc., Blacksburg, VA 24060, USA; davism@lunainc.com

**Keywords:** distributed fiber optic sensing, structural health, rural bridges, fiber Bragg grating sensing

## Abstract

Fiber-reinforced polymer composites are an excellent choice for bridge decks due to high strength, lightweight, resistance to corrosion, and long-term durability with a 100-year design life. Structural health monitoring is useful for the long-term assessment of the condition of the bridge structure and obtaining a response to complex loads considering environmental conditions. Bridge structures have been studied primarily using distributed fiber optic sensing, such as Brillouin scattering; however, critical events, including damage detection, can be missed due to low spatial resolution. There is also a critical need to conduct a comprehensive study of static and dynamic loading simultaneously for fiber-reinforced composite bridge structures. In this study, a novel approach was implemented using two sensor technologies, optical frequency domain reflectometry and fiber Bragg grating-based sensors, embedded in a glass-fiber-reinforced composite bridge deck to simultaneously monitor the deformation response of the bridge structure. The optical frequency domain reflectometry sensor utilizing Rayleigh scattering provides high spatial strain resolution were positioned strategically based on expected stress distributions to measure strain in the longitudinal, transverse, and diagonal directions along the span of the composite bridge. Furthermore, fiber Bragg grating based sensors are used to monitor the response to dynamic vehicular loading and deformations from an automotive-crash-type event on the bridge structure. To monitor environmental variables such as temperature, a custom wireless configured sensor package was developed for the study and integrated with a composite bridge located in Morgan County, Tennessee. Additionally, a triaxial accelerometer was used to monitor the vehicular dynamic loading of the composite bridge deck in parallel with fiber Bragg grating sensors. When appropriate, mid-point displacements were compared with strain-distribution measurements from the fiber optic sensor-based data.

## 1. Introduction

Structural health monitoring (SHM) utilizing distributed fiber optic sensing (DFOS) is essential for overseeing the long-term health of structures, including detecting damage locations, lowering maintenance costs, and extending the service lives of, for example, bridges [1,2]. Such transportation structures are subjected to bending moments, transverse stress, and shear loadings, which can result in stress concentrations not easily detectable during visual inspections [3]. Additionally, real-time monitoring provides on-demand measurements of variables, such as vehicular loads, through a variety of tools, wireless accelerometers, and magnetometers [4]. Fiber optic sensing (FOS) is an effective technique for such continuous monitoring. It can be used to examine composite deck panels in bridges, to assess the structural health of such panels, and to detect damage locations throughout the life of a bridge [1].

Indeed, FOS has been used extensively for various structural applications to monitor strain and thermal loads [5,6,7,8,9,10,11,12]. This has included the use of fiber Bragg grating (FBG) sensors, which are used for discrete sensing regions and are extremely useful for monitoring the response from dynamic events, including strain measurements [13,14,15]. Similarly, high-definition fiber optic sensors (HD-FOS) consisting of low-cost lightweight continuous optical fibers based on Rayleigh scattering affords high spatial resolution, with thousands of measurement points along the sensors. Moreover, it has immunity to electromagnetic interference and is flexible and easy to embed in harsh environments, such as the environment during composite manufacturing. Further, HD-FOS is based on optical frequency domain reflectometry (OFDR), which consists of swept-wavelength interferometry that utilizes a tunable laser source (TLS). When a change in strain or temperature occurs in a fiber, a local proportional change in the Rayleigh scatter pattern is reflected along the fiber’s profile. This allows the strain or temperature state to be known spatially along the fiber optic sensor [16,17,18]. This is more advantageous than strain gages, which require the installation of several arrays and are most useful if the locations of stress concentrations are known in advance, unlike with HD-FOS [19].

Large-scale structures such as bridges require significant economic investment and are closely connected to vehicular safety. Bridges are susceptible to fatigue loads, environmental effects, and crack deformations that can compromise the structural integrity of a structure [5]. Fiber-reinforced polymer composites (FRPs) are attractive for civil infrastructure, including bridge decks [20,21]. FRPs offer high specific strength and stiffness and are lightweight, inexpensive, and resistant to environmental degradation. FRP deck panels address a critical need for durable and long-lasting bridges and other civil infrastructures that are often subjected to various loads from routine vehicular traffic.

However, as bridges are integral to transportation infrastructures, FRPs are subjected to mechanical loads and can exhibit complex dynamic behaviors. It is thus critical to monitor the parameters of such materials in various component form. For DFOS, the three scattering processes that may occur are Raman, Brillouin, and Rayleigh [1,22,23]. Raman scattering, which has an intrinsic dependence on temperature, has been utilized for civil applications, such as detecting water leakage in dikes [1,22,24]. Brillouin-based DFOSs, which has intrinsic dependence on temperature and strain, have been extensively used in civil engineering structural applications due to their long-range measurement potential to monitor the global behavior of structures, including small concrete bridges and long-span bridges [1,22,25,26,27,28,29]. However, Brillouin DFOS has a lower spatial resolution compared to Rayleigh OFDR—although the latter is limited to shorter measurement lengths [1,16,22,30,31]. Despite this drawback, Rayleigh scattering has a high density of sensing points, which is advantageous in terms of “filling a range of application not easily covered neither by Brillouin nor by and Raman based DFOSs” [22]. Siwowski et al. utilized Rayleigh OFDR to monitor all-FRP composite bridge structures measuring the strain and deflection profiles for static-loading and dynamic-loading cases [32,33]. FBG-based sensors have been successfully embedded in concrete to monitor strain during the construction of Streicker Bridge, a pedestrian bridge at Princeton University, in parallel with a Brillouin-based FOS, which was also deployed for this structure. The FBG was utilized to validate the early-age behavior of concrete to identify damage events, such as induced cracking [34]. Additionally, FBGs have been used to monitor strain and temperature signals for fatigue-prone welding joints in an orthotropic steel deck arch bridge [35]. Gerbremichael et al. used FBGs to monitor an all-FRP composite bridge for controlled loading, measuring quasi-static and dynamic strains within less than 10 με [36]. Miśkiewicz et al. used different systems of sensors, including FBGs, to measure strain and temperature changes for an all-FRP composite 14 m span footbridge [37]. However, there is a critical need to monitor static and dynamic loading simultaneously on demand for FRP composite bridge structures. Utilizing a Rayleigh OFDR signal for the high-spatial-resolution monitoring of static loading and FBGs for monitoring dynamic loading show promise as novel viable solutions for capturing the structural health of bridge structures. In this connection, wireless sensor networks (WSNs) are utilized for civil structural applications and contain sensor nodes that can be deployed over a structure to monitor its health in real time. The benefits include reduced installation costs, rapid deployment, and a reduced chance of data-logging failure compared to wired sensor networks [38,39]. Furthermore, wireless technology for SHM affords the potential to capture bridge deformation and effectively provide diagnostics of a structure’s health. WSNs have also been used for cable-style bridge structures, steel, and concrete bridges and have also been used to measure acceleration, velocity, temperature, and displacement [40,41,42,43,44,45,46,47,48,49,50].

In this work, the authors introduce for the first time monitoring the static loads using HD-FOS and dynamic loads using FBGs of an FRP deck panel subjected to vehicular loading as a viable novel solution to effectively address the structural health monitoring of fiber-reinforced polymer composite (FRP) bridge structures, in particular an FRP deck panel for spatial strain distribution using HD-FOS subjected to static loading. The strain development between the HD-FOS and FBG sensors used simultaneously to measure strain when the FRP deck panel is subjected to vehicular loading for a dynamic loading condition are compared. Additionally, a novel scheme using a wireless sensor network for vehicle-detection measurements evaluating the performance and dynamic response using wireless accelerometers, and environmental conditions, such as the temperature of the FRP bridge deck system, are demonstrated using a visual interface optimized for cloud computing and display. The evaluation and performance of the smart composite deck was conducted in Morgan County, Tennessee, USA, with participation from the Tennessee and Florida Departments of Transportation.

This work is divided primarily into three areas. The first is utilizing HD-FOS for static loading and FBGs for dynamic loading to evaluate deformations of FRP polymer composite deck panel prior to installation at bridge site. The key advantage is the unique capability to evaluate static loads with high spatial resolution in longitudinal, transverse, and diagonal directions simultaneously along the span of an FRP composite deck panel. In parallel, high-speed measurements using an optical sensing interrogator and FBG are used to measure dynamic and absolute strain response along the top of the composite deck panel. The uniqueness of the FBG is the capacity to capture dynamic vehicular events such as crash-type events on the composite bridge structure. Secondly, after the installation of composite deck panels at the bridge site, one panel is evaluated measuring absolute strain response to vehicular loading (a dump truck) traveling over the span of the bridge structure for both static and dynamic loading case studies. Lastly, a dynamic case study evaluates the accelerometer response to vehicular loading traveling over a composite deck panel. The combination of HD-FOS and FBG affords the ability to monitor the deformation and dynamic responses simultaneously in real-time traffic conditions. Fiber optic sensing provides very high spatial resolution using HDFOS sensors and time resolution using FBG sensors. However, to acquire data, suitable interrogators have to be physically connected to these sensors in a wired mode due to the current limitations of this technology not supporting wireless data acquisition. In this research, we developed the concept of using wireless sensors to alert when fiber optic sensors need to be interrogated on-demand in response to environmental events (thermal and humidity excursions coupled with mechanical response) and extreme events, such as crashes on the bridge detected by accelerometers.

## 2. Sensing Principles of HD-FOS and FBGs

Two different methods were used to interrogate the optical fiber sensors and will be described within this section: OFDR, which takes advantage of the Rayleigh scatter within the entire fiber to provide a high-spatial-resolution measurement, and a method that uses FBGs to reflect a measurement-dependent specific wavelength at a discrete point on the fiber.

### 2.1. Optical Frequency Domain Reflectometry (OFDR)

**Figure 1:**
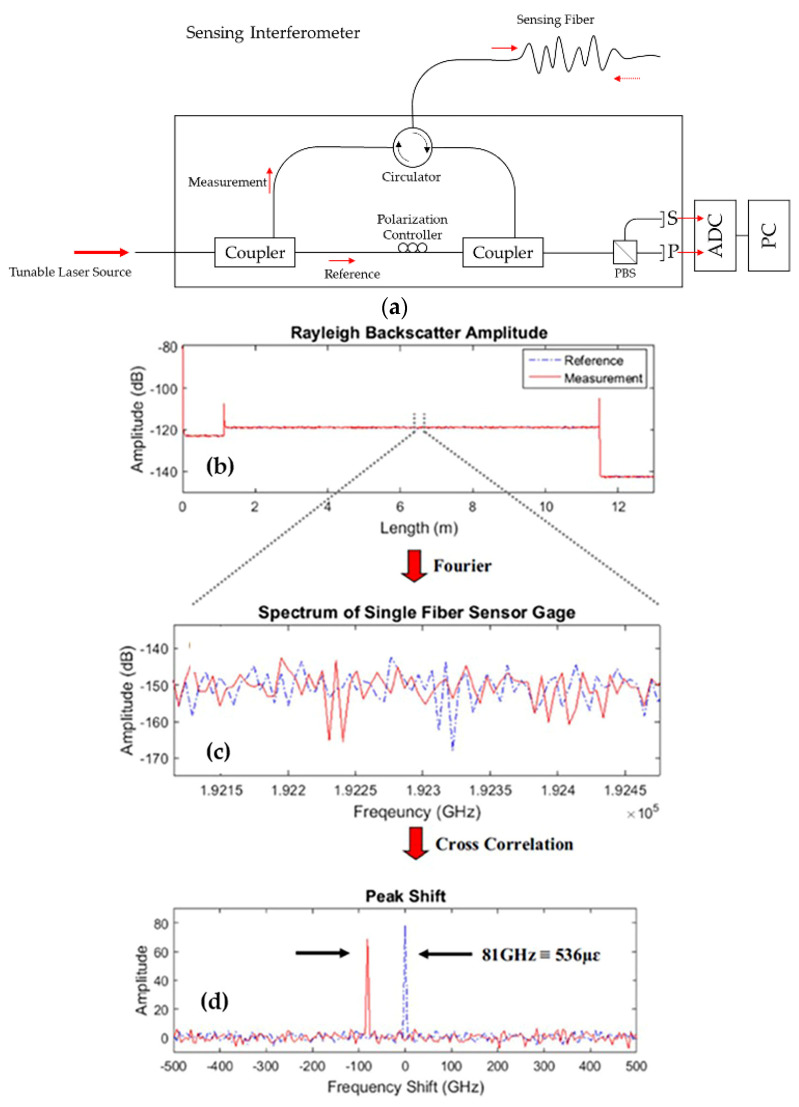
(**a**) Schematic of the optical frequency domain reflectometry (OFDR) principle. (**b**) Example Rayleigh scatter measurement with reference (unstrained) and measured strain values induced from external stimuli. (**c**) An inverse Fourier transform is applied to a gage-length section of the Rayleigh scatter traces. (**d**) A cross-correlation of the reference measurement spectrum and the strain-measured spectrum is performed, resulting in a peak shift between the two spectra, reprinted with permission from Refs. [17,18].

OFDR (Figure 1a) is an interferometric technique that takes advantage of the Rayleigh scatter naturally exhibited by most optical fibers. Rayleigh scatter arises when light interacts with the random and micro-scale variations in the refractive index that exist along the length of the fiber. These variations are permanent features locked into the glass matrix. As these variations are random, they are uncorrelated with one another, and the spectral reflection signature from any region of the fiber uniquely identifies that location. When the optical fiber is subjected to a change in strain or temperature, there is an induced shift in the spectral signature produced by these Rayleigh scatter locations.

Changes in strain (or temperature) are calculated by comparing the spectral content of a scan of the fiber during measurement with the spectral content of a reference scan that was taken when the fiber was experiencing baseline or neutral conditions. A tunable laser source is used to acquire measurements, and the raw data are a function of the laser’s instantaneous wavelength. These data are processed to produce a response that is a function of delay (position along the length of the fiber). These delay-domain data exclude the Rayleigh scatter contributed by optical components external to the device being tested. It is the delay-domain data that are manipulated to obtain measurements of strain and temperature. To determine the change of the measurement at a particular location, the delay-domain data around that spot are windowed (the specified gage length determines the width of the window), and an inverse Fourier transform is performed to calculate the spectral content of that region. These selected data from the measurement scan are cross-correlated with the spectral data from the same region on the reference scan. Figure 1b shows the spectral content of a 5 mm fiber segment. The dotted line is the reference data, and the solid line is the data acquired after the fiber had been strained (Figure 1c). Figure 1d shows the cross-correlation result.

The spectral shift determined by the cross-correlation operation is converted to strain or temperature change using a calibration coefficient, or gage factor. By repeating this calculation for points along the length of the sensor, a distributed measurement is made. The ODiSI 6104 HD-FOS incorporates a reflection-suppressing termination and angled physical contact (APC) connectors. It has the ability to measure a strain value range of ±15,000 με, making it suitable for monitoring flexural structures subjected to mechanical and thermal loads [16,17,18,51,52,53,54].

### 2.2. Fiber Bragg Grating Measurements

**Figure 2:**
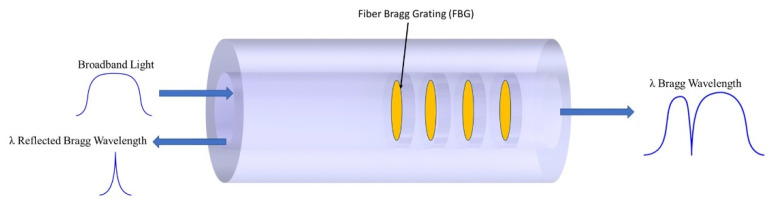
Illustration of the FBG principle.

An FBG is an optical filtering device consisting of localized periodic modulation of the refractive index present in the core of an optical fiber, reflecting light of a certain wavelength created by a laser-inscription process, as shown in Figure 2. As broadband light is propagated along the fiber core at a particular wavelength, satisfying Bragg’s interference condition, the light is reflected back, while the light not in resonance with this condition is transmitted with a small amount of attenuation [55,56,57,58]. FBGs are sensitive to environmental conditions, including strain and temperature. When the FBG is subjected to axial strain, the Bragg grating period will change, resulting in a shift in the Bragg wavelength [58]. This coupled strain and optical effect on the grating structure corresponds to the refractive index changes and grating spacing [55,59].

Measurements of the reflected wavelength are made using Luna’s Hyperion si155 instrument, a swept wavelength laser-based interrogator. By using a reference to know the absolute wavelength of the light transmitted into the fiber as a function of time, the respective reflected wavelengths from the FBGs written into the fiber and received by the photodetectors are known, resulting in a very accurate determination of each FBG’s optical wavelength. The change in the FBG from its original state is converted into the measured strain or temperature. The total strain of the FBG is governed by the following equation:

(1) $$\epsilon_{Total}=10^{6}\left[\frac{{\left(\frac{\Delta \lambda }{\lambda_{0}}\right)}_{s}-{\left(\frac{\Delta \lambda }{\lambda_{0}}\right)}_{T}}{F_{G}}\right]+\frac{{\left(\frac{\Delta \lambda }{\lambda_{0}}\right)}_{T}}{S_{T}}CTE_{T},$$

where *λ* represents the optical wavelength shift for both the strain sensing and thermally induced changes.

*F_G_* denotes the gage factor of the optical strain gage and is expressed in the following form, with the strain-sensing FBG measured in units of microstrain per normalized wavelength shift:

(2) $$\frac{\mu \epsilon }{{\left(\frac{\Delta \lambda }{\lambda_{0}}\right)}_{s}}$$

The temperature compensator, *S_T_*, represents the temperature compensator thermal response and is expressed in the following form:

(3) $$\frac{{}^{\circ}C}{{\left(\frac{\Delta \lambda }{\lambda_{0}}\right)}_{T}}$$

The coefficient of thermal expansion, *CTE_T_*, signifies the thermal expansion of the temperature compensation gage and is represented in the following form [60,61]:

(4) $$\frac{ppm}{{}^{\circ}C}$$

## 3. Materials and Methods

### 3.1. FOS and FRP Deck Panels

**Figure 3:**
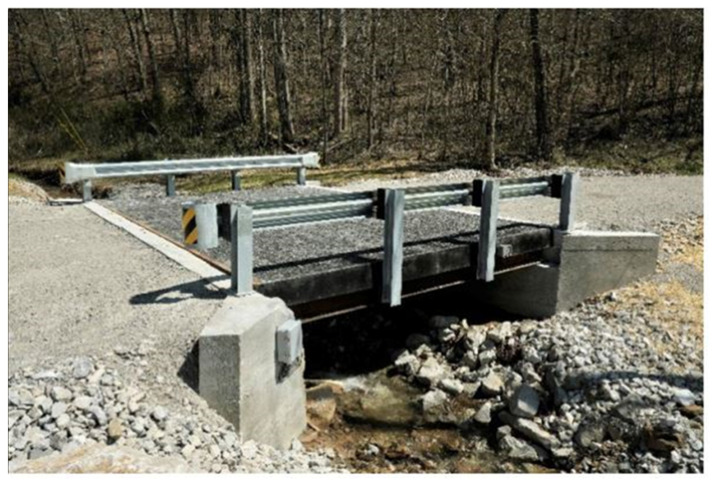
Photograph of the installed fiber-reinforced polymer composite bridge supported by steel girders.

**Figure 4:**
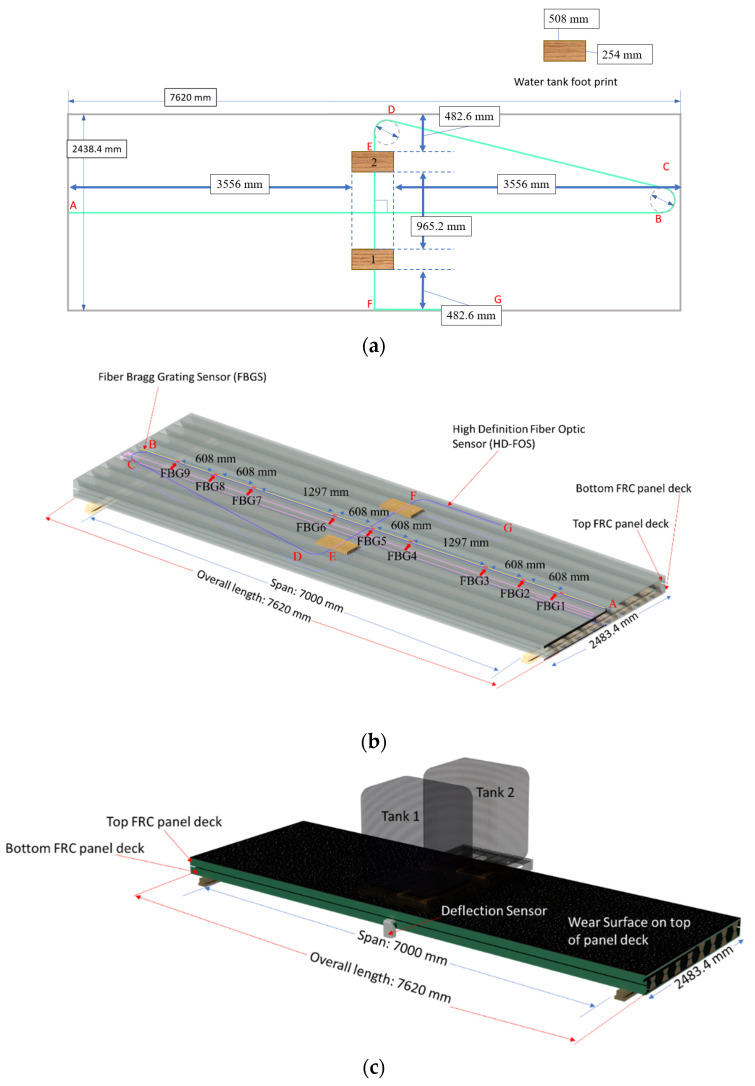

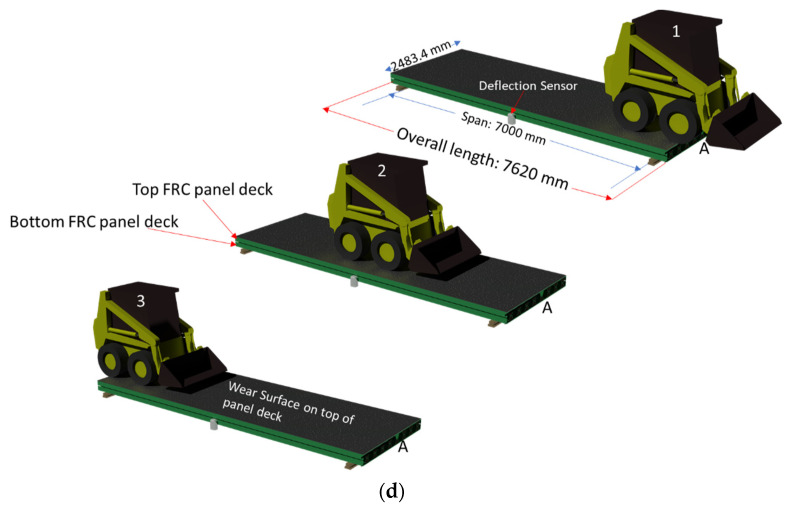
**Static and dynamic loading of deck panel:** (**a**) Top isometric view of HD-FOS sensor configuration and water tank footprint (**b**) Isometric view of the HD-FOS and FBG sensor configurations and water tank footprints on the composite deck panels, respectively. (**c**) Static loading schematic representation on the FRP deck panel. (**d**) Dynamic loading schematic showing vehicle travel from the edge of FRP deck panel (position 1) to the center (position 2) and to the opposite end of the FRP panel (position 3). Note: FRP and FRC both refer to the fiber-reinforced polymer composite deck panel.

A concrete-deck-based bridge was replaced with a glass FRP bridge deck in this study as a demonstration project, as shown in Figure 3. The strain response of the FRP bridge was monitored using an SHM application that incorporated two optical sensing interrogator instruments from Luna Innovations, Inc. (Blacksburg, VA, USA; summarized in Table 1, Table 2 and Table 3). The first instrument was the commercial multichannel ODiSI 6104 with HD-FOS and was utilized for high-spatial-resolution strain measurements along the top and bottom of the bridge’s deck panels (Figure 4a,b). The second instrument was the commercial high-speed measurement optical sensing interrogator (Hyperion si155) utilized to record measurements from the FBG sensors. These captured dynamic and absolute strain measurements along the top of the deck panel, as shown in Figure 4b [62].

**Figure 5:**
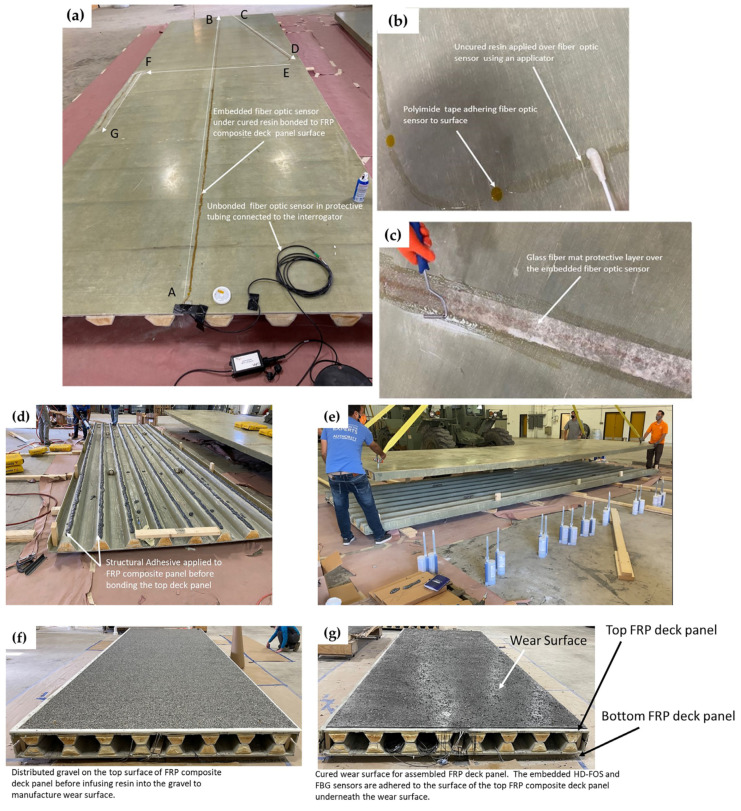
**Assembling of FRP deck panel and integration of fiber optic sensors (FOS):** (**a**) Example installation of the high-definition fiber optic sensor (HD-FOS) sensor layout bonded to the surface of the FRP deck panel with cured resin. (**b**) Higher-resolution image of the fiber optic sensor bonding to the FRP deck panel. The sensor is held in place using circular polyimide tape while the resin is applied to allow curing. (**c**) A glass fiber mat is bonded on the embedded fiber optic sensor to further protect the fiber optic. (**d**,**e**) Adhesively bonding the upper and lower FRP composite deck panels. (**f**,**g**) Applying wear surface.

Figure 5 shows an example installation of one of the FRP panels, including the sensor layout for the HD-FOS. The FBG sensory layout procedure is not shown; however, the sensor was installed with a similar protocol. Two full-scale FRP panels were manufactured using vacuum-assisted resin transfer molding (VARTM) by Structural Composites in Melbourne, FL, USA. The top and bottom of the panels were sanded using 220 grit paper, vacuumed, and then wiped with acetone, using cloths to remove debris. This ensured the sensors bonded sufficiently to the deck panel surfaces. The HD-FOS were polyimide-coated glass fibers 155 μm in outer diameter; they were bonded to each side of each deck panel for a total of two sensors in the configuration shown in Figure 4a and Figure 5a–c.

The measured spectral shift (GHz) from the ODiSI-6104 interrogator for HD-FOS is converted to determine strain-distribution values based on the following polynomial relation:

(5) $$\varepsilon \left(v\right)=ax+bx^{2}$$

where *x* is the spectral shift; *v* denotes the optical frequency, and *ε* denotes the strain (μm/m). Table 4 summarizes the coefficients *a* (με/GHz) and *b* (με/GHz^2^) used to relate the spectral shift, *x*, and the strain, *ε*, in Equation (5). It must be noted that increases in strain values are indicated by negative coefficient values [8]. A data-acquisition rate of 2.1 Hz and a gage pitch of 2.6 mm was used to measure the strain-distribution response using the ODiSI-6104 interrogator unit.

A Draw Tower Grating FBG (DTG-LBL-1550-125, FBGS, Geel, Belguim) was inscribed into organic modified ceramic (ORMOCER^®^)-coated glass fiber, measuring 195 μm in outer diameter. According to FBGS, the typical strain sensitivity is 7.8 με^−1^ × 10^−7^ [65]. In this study, the FBG was directly bonded onto the composite deck panel’s top surface, and FBG temperature compensation was not used due to potential voids and delamination development under the topcoat. Removing temperature compensation terms from Equation (1), the total strain was calculated using the following relation:

(6) $$\epsilon_{Total}=10^{6}\left[\frac{{\left(\frac{\Delta \lambda }{\lambda_{0}}\right)}_{s}}{F_{G}}\right]$$

Prior to bonding FBG to the composite deck panel, the FBG gage factor was calculated, based on measuring the optical frequency shift corresponding to an induced strain based on Equation (6). The induced strain ranged from 0 to 12,000 με, where a strain was applied to the FBG with a peak of 12,000 με and unloaded back to 0 με in displacement steps of 2000 με. A mean gage factor of 0.775 was calculated for the FBG sensor and had a sensitivity of 1.2 pm/microstrain. The strain-distribution response was collected at a data acquisition rate of 1000 Hz using the Hyperion si155 interrogator. Figure 4b shows the configuration of the sensor layout and the locations of the nine FBGs on the top surface of the composite deck panel.

Unbonded or unsecured free lengths of HD-FOS and FBG sensor sections were then carefully coiled into an enclosure. An epoxy resin (M-bond AE-10, Micro-Measurements, Wendell, NC, USA) was used for bonding the sensors along the segments of interest shown in Figure 4a,b. The resin was cured for approximately 24 h. Additionally, a chopped glass fiber mat was bonded over both sensor types as a protective layer, as shown in Figure 5c.

Following adhesively bonding the panels (Figure 5d,e) a wear surface was applied to the top side of two of the deck panels as shown in Figure 5f,g. This wear surface was manufactured using a co-cure resin combination with aggregate to produce a tough, durable wear surface for the top of the FRP bridge deck. Co-Cure is a polyester-polyurethane hybrid coating described in U.S. Patent #9,371,468 based on a polyester crosslinking reaction, and the polyurethane chain extension reaction occur concurrently during the cure [66]. The Co-Cure 20 contains 80% polyester and 20% polyurethane. The polyurethane is a two-part system comprising a polyester polyol resin and a polymeric isocyanate. Thus, Co-Cure contains four components: polyester resin, polyol, isocyanate, and methyl ethyl ketone peroxide (initiator). Changing the ratios of the CoCure resins offers strain adjustability. For the bridge deck, the polyester/polyol were blended together at the factory at a 80/20 ratio and designated CC20C01BB1793E. The formulation used for mixing onsite was 100 parts by weight (pbw) CC20C01BB1793E, 15 pbw CC-ISO-B-EXE (Isocyanate), and 1.5 pbw Norox MEKP-925 (methyl ethyl ketone peroxide). The aggregate was spread evenly over the bridge deck surface to a depth of 12.7 mm (Figure 5f), then the Co-Cure 20 was poured over the aggregate to give the surface better grip; the aggregate was sprinkled over the Co-Cure 20, leaving some of the aggregate surface exposed (Figure 5g). The wear surface was allowed to cure overnight at room temperature.

It is noteworthy that the coupled HD-FOS and FBG’s detection of localized strain changes presents an advantage in the early detection of damage, including cracks in the laminate of the FRP deck panels. Prior to installing the composite bridge deck on the field, laboratory static and dynamic loadings were performed for the decks.

Prior to installation at the bridge site and being adhered to girders, a composite deck panel was elevated approximately 200 mm aboveground, as shown in Figure 4c. The panel was evaluated for strain distribution and subjected to vertical static loading of up to 19.6 kN at its mid-span using two water tanks, i.e., 9.8 kN loading based on an American Association of State Highway and Transportation Officials (AASHTO) standard [67,68,69]. The panel was subsequently subjected to dynamic loading (Figure 4d) using a vehicle of about 27 kN by driving the load across the deck panel end-to-end at approximately 0.44 m/s. The tension and compressive strain distribution that resulted from the static and dynamic loading were monitored using HD-FOS with a gage pitch of 2.6 mm and an acquisition rate of 2.1 Hz. Additionally, the compressive strain distribution that resulted from the dynamic loading was monitored by simultaneously using the FBG sensor and HD-FOS at an acquisition rate of 48 Hz.

**Figure 6:**
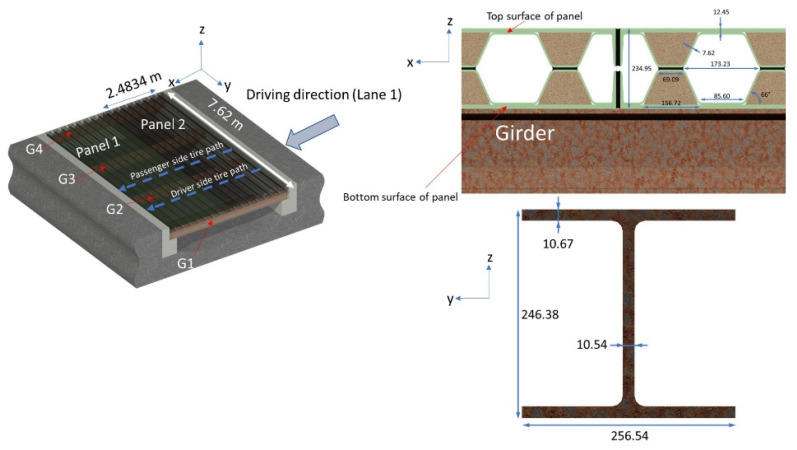
(**Left** image) Schematic representation of composite deck Panels 1 and 2, where strain and dynamic loadings were measured on FRP Panel 1. G1–G4 represents girders 1 to 4. (**Right** image) Cross-sectional view of upper and lower half segments of bonded composite deck panel and girder. Note: Units in right image are in millimeters.

**Figure 7:**
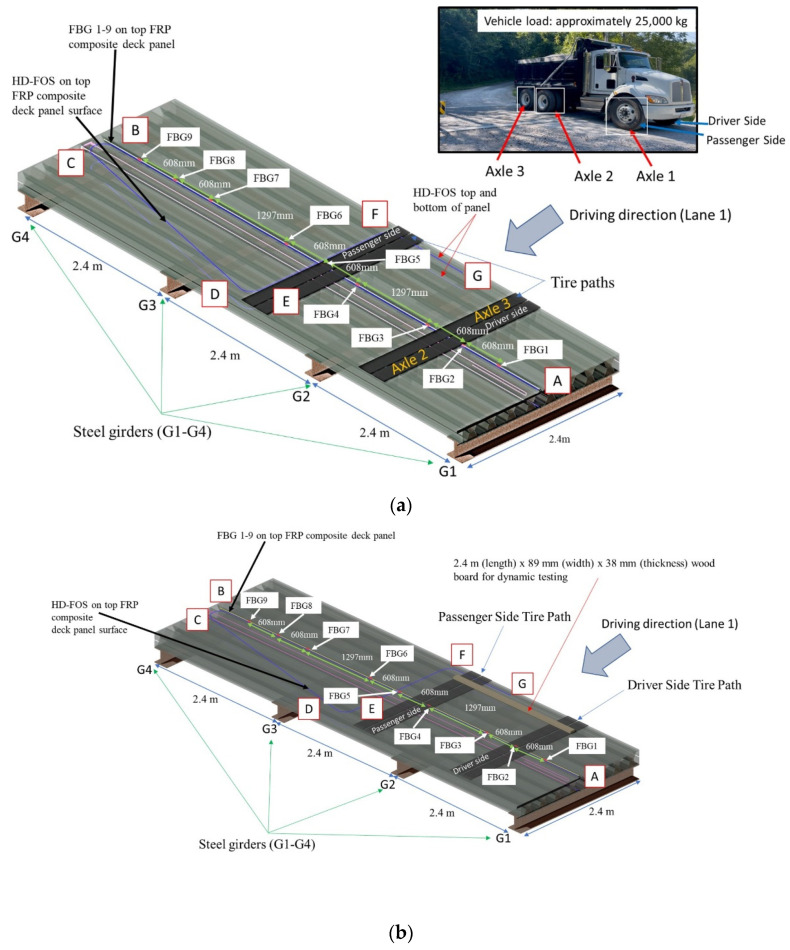
**Static and dynamic loading cases of FRP deck panel:** (**a**) Static loading case of a vehicle with axle 2 (X2) and axle 3 (X3) positioned on FRP deck Panel 1 to measure the strain response of HD-FOS on the top and bottom surfaces of the deck panel. (**b**) Dynamic loading case wherein the wooden board is placed to amplify the detection of strain response from FBGs on the top surface of FRP deck Panel 1.

Following the confirmation of the strain profile and the accuracy of the HD-FOS and FBG sensors, the two FRP deck panels, denoted as Panel 1 and Panel 2 (as shown in Figure 6), were installed on the field in less than a day. This site was selected to replace a concrete bridge with poor structural support. As shown in Figure 7, an approximately 25,000-kg vehicle with 3-axle wheels, denoted as axle 1 (X1), axle 2 (X2), and axle 3 (X3), was used to monitor the strain response of the FRP deck panels. It must be noted that only Panel 1′s strain response is evaluated and discussed in this study. Additionally, “A” denoted in figures throughout this study signifies the datum reference point for both the HD-FOS and FBG segment “AB” (Figure 4b and Figure 7a) in the sensor layout on the panel deck.

### 3.2. Wireless Sensing Monitoring

**Figure 8:**
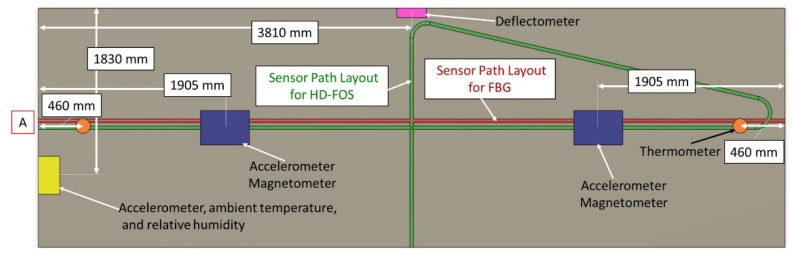
A schematic with the approximate locations of the magnetometer, accelerometer, thermometer, deflector, and relative humidity wireless module setup for the static loading of FRP Panel 1. Point “A” denotes the datum reference for the sensor path layout for both HD-FOS and FBG on the panel deck.

In addition to the fiber optic sensing technologies, HD-FOS and FBG, a custom wireless sensing network was designed to monitor thermal excursions and crash-type events for the FRP composite bridge. The FRP composite bridge was equipped with 15 sensors and a synchronized video feed integrated into a wireless network. After local conditioning and compression, the data was sent to a cloud host where it was archived and made available for live monitoring or historical analysis with custom Web-based tools. The wireless sensor array consisted of two three-axis accelerometers for registering applied bridge forces and vibrations, two three-axis magnetometers for counting vehicles, eight composite digital thermometers embedded for measuring material temperatures and differentials, one ambient hygrometer, one thermally isolated ambient thermometer, and one Hall-effect non-contact absolute deflection sensor for measuring absolute bridge deck flex in fractions of a millimeter. The Hall effect deflection sensor is an inexpensive way to identify overweight conditions using measurement of relative bridge movements to derive an indirect measurement of vehicle weight. A simplistic schematic representation of their locations is shown in Figure 8.

The data was obtained at 20 mS intervals, timestamped, and recorded locally. Data reduction and analysis algorithms were then run on a bridge-resident central processing unit (CPU) to select and compress the data in order to accommodate the available bandwidth of the internet link and the database intake rates of the Amazon Web Services (AWS)-hosted MongoDB. Original timestamps from the sensors are preserved through the process, so archived data remains time-coherent with other sensors and with the AWS-resident video stream, enabling detailed past incident analyses. The accumulated data can be viewed live or recalled indefinitely from a Web browser utilizing a custom dashboard built specifically for this project. A custom dashboard was designed to handle the unique characteristics of the bridge data that render it difficult to analyze. The data is normally a continuous stream of sparse data, such as temperature and vehicle count (10 s period in a single packet), but is mixed with spikes of intense and highly detailed events, such as acceleration envelopes (20 mS period in 100 packets or greater bursts) from a vehicle’s passage.

The bridge was in an extremely rural location, which presented a challenge to moving volumes of data at low latency. Neither long-term evolution (LTE) cellular nor commercial internet was available at the time of installation. To provide connectivity, the system was designed to use the new CAT-M1/NB-IOT narrowband cellular radio network provided by major carriers for telemetry/IoT applications requiring extended reach and reliability. High-speed fiber access was added later in order to carry full videos, and the sensor network was modified to select either network in an automatic failover configuration. For rural areas, the CAT-M1 system acts as a primary system. In areas with no internet access whatsoever, data is always logged locally for manual pickup from a portable wireless device, such as a laptop or a smartphone. The system reports system diagnostics, such as CPU/modem temperatures, signal quality, and software status to servers, as well as accepts supervisory commands from the Web console for remote configuration, maintenance, and upgrades, to minimize expensive site visits. Sensor nodes communicate on an independent wireless network to allow the system to tolerate individual sensor failures, as well as manage sensor network traffic loads. A third wireless network hosts local administrative and control functions, as well as manual “drive-by” data pickup. These isolated local networks enhance reliability and serve to secure the bridge systems from the internet and hackers. Once the FRP deck panel was installed with the girder supports, a vehicle with approximately 25,000 kg of loading was driven across the panel up to approximately 2 m/s to measure the accelerometer’s responses.

## 4. Results and Discussion

Given the complexity of the deformations of the FRP deck panel when loaded statically and dynamically, the panel was evaluated for two cases. The first case is loading the FRP deck panel without girder support prior to installation at the bridge and the second case is loading the panel with girder support (after installation at the bridge site) for the following proposed experimental program:

(1) monitor the OFDR signal obtained from the HD-FOS strain response and mid-span displacement for a statically loaded FRP deck panel without girder support;
(2) monitor strain response comparison between HD-FOS and FBG measured simultaneously when the FRP deck panel is subjected to vehicular loading without girder support;
(3) measurement of strain response signal from both sensor types while the vehicle (dump truck) is traveling over the FRP panel with girder support, when subjected to both static (HD-FOS) and dynamic (FBG) loading cases;
(4) measurement of accelerometer signal response of panel subjected to vehicular loading. This measurement was observed in parallel to a vehicle (dump truck) traveling over the FRP panel to couple with FBG signal response;
(5) measurement of a temperature response signal from digital thermometers embedded within the top and bottom FRP deck panels to monitor thermal excursions within the deck panels.

### 4.1. Static Loading and Dynamic Loading of FRP Decks without Girder Support

**Figure 9:**
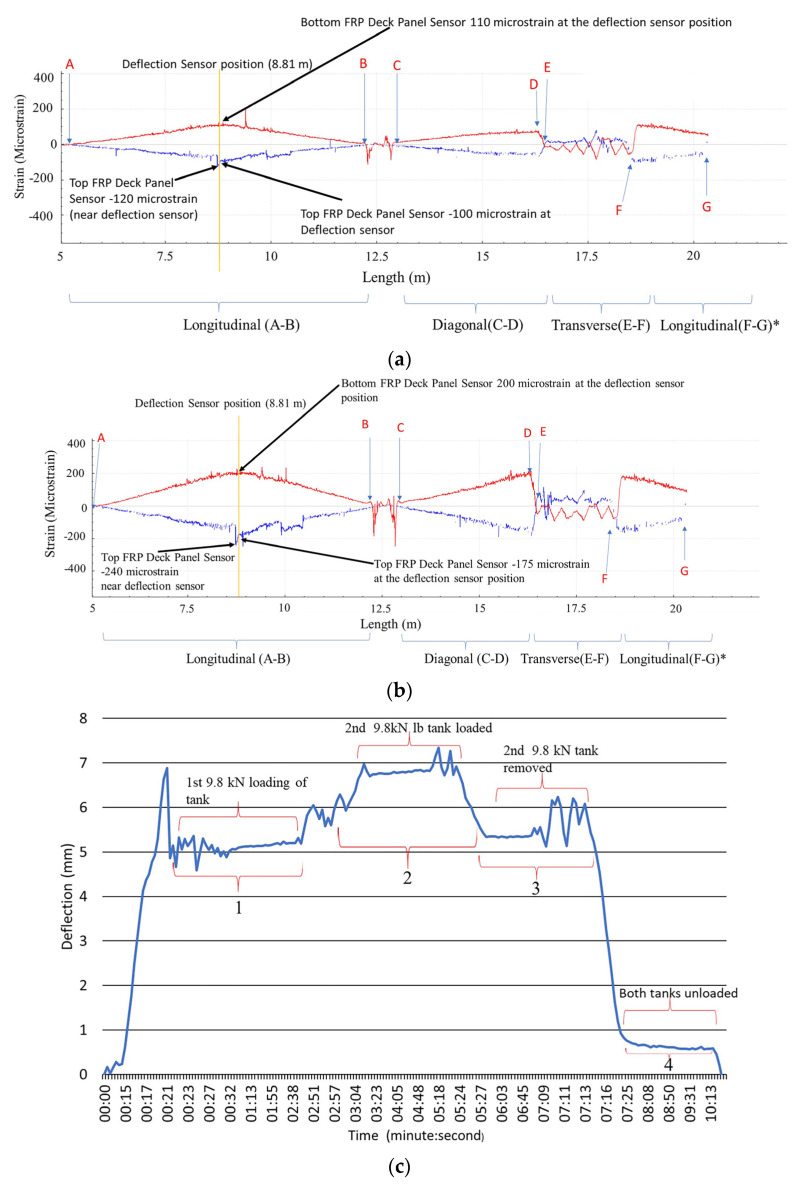
The spatial strain distributions of the FRP deck panel with the HD-FOS, subjected to (**a**) 9.8 kN and (**b**) 19.6 kN of static loading. The blue curves are the strain response from the top FRP deck panel, and the red curves are the strain response from the bottom FRP deck panel. Longitudinal segment A to B, diagonal segment C to D, transverse segment E to F, and the longitudinal segment F to G* represents the various sections of HD-FOS measured for strain distribution. (**c**) The corresponding displacement at the mid-span of the deck panel. Segment 1 corresponds to the loading of the 1st water tank (9.8 kN) on the FRP deck panel. Segment 2 corresponds to the loading of the 2nd water tank (9.8 kN) for the combined loading of 19.6 kN on the FRP deck panel. Segment 3 corresponds to the removal of 9.8 kN water tank leaving a static loading of 9.8 kN on FRP deck panel. Segment 4 corresponds to the removal of the last water tank (9.8 kN) where both water tanks are removed.

Figure 9 shows the strain distributions of the HD-FOS for the various segments on the FRP deck panels, in response to the static loading of two water tanks of approximately 9.8 kN each without girder support. The longitudinal segments AB, the diagonal segment CD, and the longitudinal segment FG showed progressive increases in strain as the static load was applied, as shown in Figure 9a,b. For segment AB, both loading conditions of the water tanks exhibited larger tensilestrains (absolute values) at the deflection sensor mid-span point location corresponding to the bottom deck panel compared to the top deck panel, corresponding to compressive strains. For example, the peak strain observed for the loading of the first water tank for the top deck panel (100 με compression) was approximately 8.7% lower than the bottom deck panel peak strain (110 με tension), as shown in Figure 9a. Similarly, as shown in Figure 9b, the bottom deck panel peak strain (200 με tension) was approximately 13.3% higher than the top deck panel (175 με compression) for segment AB. The main takeaway from these results is that the bridge deck is responding in a fashion expected from the analysis. For a point load in a mid-span on the top of the bridge deck, analysis indicates that one should expect compressive strains in the top of the deck and tensile strain in the bottom, and the response should be nearly symmetric if the adhesive joint that was used to join top and bottom half-sections were responding in unison. The results in Figure 9 confirm these observations. Similarly, if we double the bending moment and shear force and the FRP bridge deck panel is in the linear elastic regime, the strains will simply be twice or near that order, given some non-linear load redistribution. Again, this takeaway was confirmed with results from Figure 9b versus Figure 9a. Notably, the transverse segment EF showed an oscillating strain pattern behavior on the bottom deck panel, unlike the top deck panel. The differences in strain could be attributed to the wear surface on the top, restricting the compressive deformation of the top panel when loaded.

The progressive deflection of the deck panel at the mid-span was measured using a deflectometer, shown for the static loads of the two water tanks (Figure 9c). The deck panel displacement increased from 5 mm, corresponding to the static load of 9.8 kN in Segment 1, to 7 mm, corresponding to the total static load of 19.6 kN in Segment 2. Similarly, the displacement decreased from 7 mm to 5 mm after the removal of one of the 9.8 kN loads in Segment 3, and after the removal of both water tanks in Segment 4, it returned to approximately 0 kN of loading.

**Figure 10:**
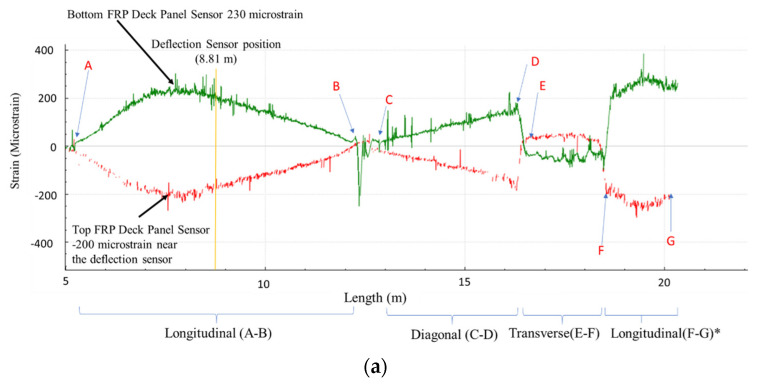

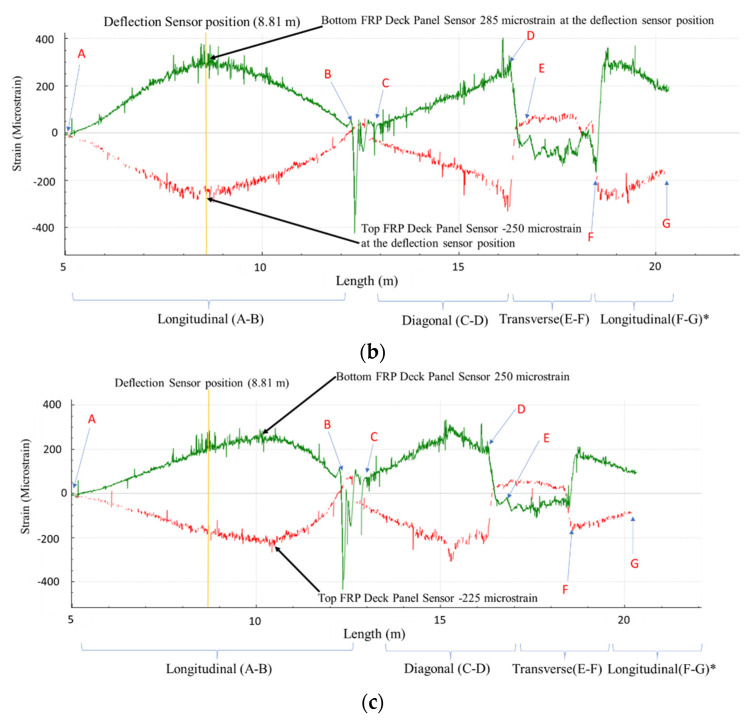
The spatial strain distributions of the FRP deck panel with HD-FOS when subjected to vehicular loading on (**a**) Position 1, (**b**) Position 2, and (**c**) Position 3 of the panel, as illustrated in Figure 4d. The red curves are the strain response from the top FRP deck panel, and the green curves are the strain response from the bottom FRP deck panel. Longitudinal segment A to B, diagonal segment C to D, transverse segment E to F, and the longitudinal segment F to G* represent the various sections of HD-FOSs measured for strain distribution.

Figure 10 shows the strain distribution of the HD-FOS for the various segments of the FRP deck panels under a vehicular load of approximately 27 kN without girder support. As aforementioned, a vehicle was driven end-to-end on the deck at approximately 0.44 m/s as illustrated in Figure 4d. The longitudinal segments AB, the diagonal segment CD, and the longitudinal segment FG showed unambiguous changes in strain distribution as the vehicle moved from the one side of the panel, Position 1 (Figure 10a), to the center of panel, Position 2 (Figure 10b) and, finally, to the opposite end of the panel, position 3 (Figure 10c). The results from Figure 10 serve to support the inference that the FRP bridge deck panel response in dynamic loading is also in agreement with the analytical solution expected from the influence line method, and having exceptionally high spatial resolution provides opportunities for isolating any manufacturing-induced defects or material defects as they will show up as singularities in strain response. As shown in Figure 10, larger tensile strains (absolute values), corresponding to the bottom deck panel, compared to the top deck panel, corresponding to compressive strains, were observed for segment AB. As an example, for Position 1 (Figure 4d), the peak strain observed for the top deck panel (200 με compression) was approximately 14% lower than for the bottom deck panel (230 με tension), as shown in Figure 10a. Similarly, as shown in Figure 10b, the peak strain for the bottom deck panel (285 με tension) was approximately 13.1% higher than the peak strain for the top deck panel (250 με compression) in segment AB for Position 2. Lastly, the top deck panel (225 με compression) peak strain observed was approximately 10.5% lower than the top bottom deck panel peak strain (250 με tension), as shown in Figure 10c. Similar to the static load, the transverse segment EF showed an oscillating strain pattern behavior on the bottom deck panel, unlike the top deck panel.

**Figure 11:**
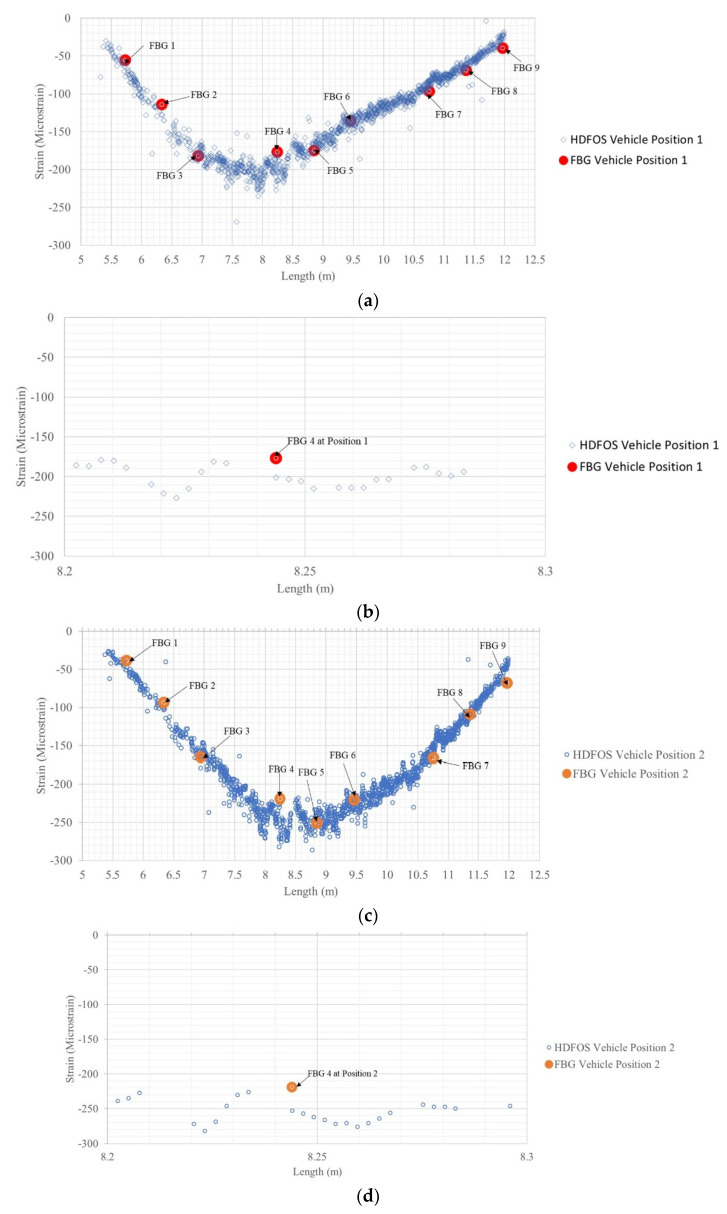

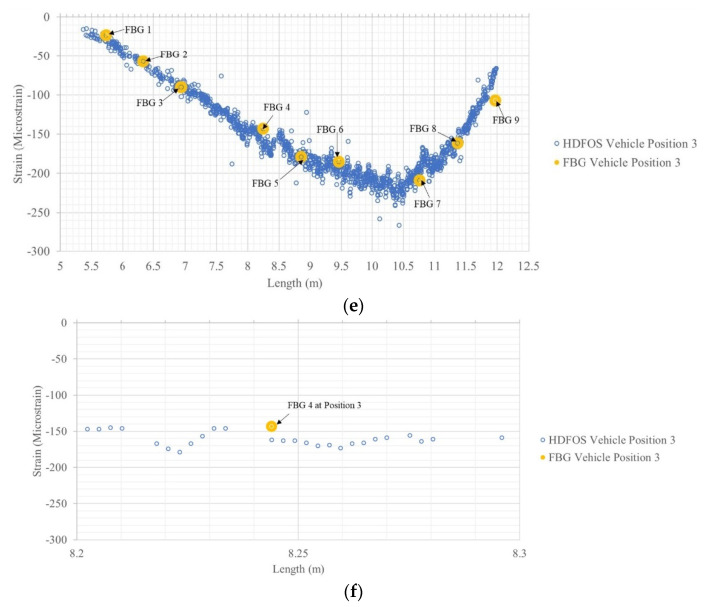
Comparison of the spatial strain distributions of the FRP deck panel between HD-FOS and FBG subjected to vehicular loading at (**a**,**b**) Position 1, (**c**,**d**) Position 2, and (**e**,**f**) Position 3 on the panel.

To compare the HD-FOS and FBG signal response, the strain distribution of Segment AB of the deck panel, corresponding to the compressive strains induced by the vehicular load, is shown in Figure 11. Segment AB represents the location where both sensor types were adhered side by side along the longitudinal segment of the sensor layout (Figure 4b). Differences in compressive strain measurements were observed between FBG and the HDFOS. For example, a difference in strain values for FBG 4 and HD-FOS is observed for the approximate sensor position 8.244 m for vehicular loading Position 1 (Figure 4d), Position 2, and Position 3, as shown in Figure 11. FBG 4 (−177 με) measured a lower (12.7%) compressive strain than the HD-FOS (−201 με) for Position 1, as shown in Figure 11a,b. Similarly, for Position 2, as shown in Figure 11c,d, FBG 4 (−219 με) measured a lower (14.4%) compressive strain value than the HD-FOS (−253 με). For Position 3 (Figure 11e,f), the compressive strain for FBG 4 (−143 με) was 12.3% lower than HDFOS (−163 με). Despite these differences in strain values, globally, the HD-FOS and FBG sensors were in good agreement qualitatively, wherein the strain values of the two sensor types visually overlapped. This is the first study that compares FBG response with HD-FOS and shows the value of integrating fiber optic sensors for bridge health monitoring. With FBGs, we have exceptional time response for dynamic loading events, while HD-FOS are very good at isolating static response without omitting critical regions of the bridge structure.

### 4.2. Static Loading and Dynamic Loading of FRP Decks with Girder Support

**Figure 12:**
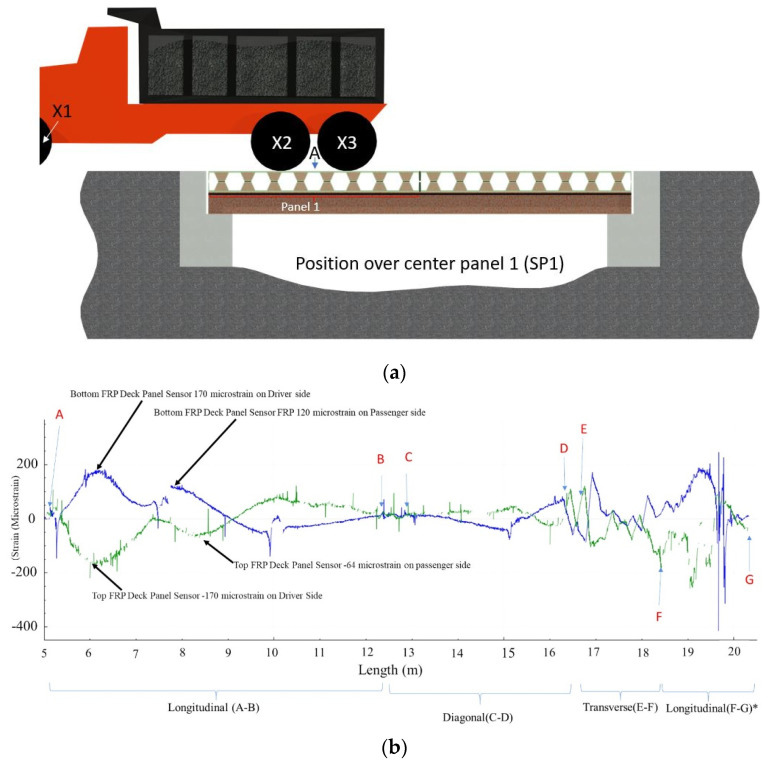
(**a**) Static loading of vehicle (SP1) centered on composite deck Panel 1 and (**b**) the corresponding spatial strain distributions of the FRP deck panel (Panel 1). X1–X3 represents the vehicle’s three axles positioned on the FRP deck panel for the static loading case. The green curve is the strain response from the top FRP deck panel, and the blue curve is the strain response from the bottom FRP deck panel. Longitudinal segment A to B, diagonal segment C to D, transverse segment E to F, and the longitudinal segment F to G* represent the various sections of HD-FOSs measured for strain distribution.

The aforementioned static loading and dynamic loading were conducted to assess the performance of the FRP deck panels. For the static loading, the vehicle’s (dump truck) axle X2 and axle X3 were positioned over the center of deck Panel 1, designated as Static Position 1 or SP1 (as shown in Figure 12a), inducing the maximum displacement of the composite deck panel. Figure 12b shows the HD-FOS strain response of the vehicle loading for the longitudinal segment AB, the diagonal segment CD, the transverse segment EF, and the longitudinal segment FG. For segment AB, two unambiguous tension and compression peaks, corresponding to the positions of the vehicle’s axle X2 and axle X3, can be clearly observed. For example, the tensile values (bottom side of the composite deck) were approximately 170 με on the driver side and 120 με on the passenger side. The compressive strain values (top side of the composite deck) were approximately −170 με on the driver side and −64 με on the passenger side.

**Figure 13:**
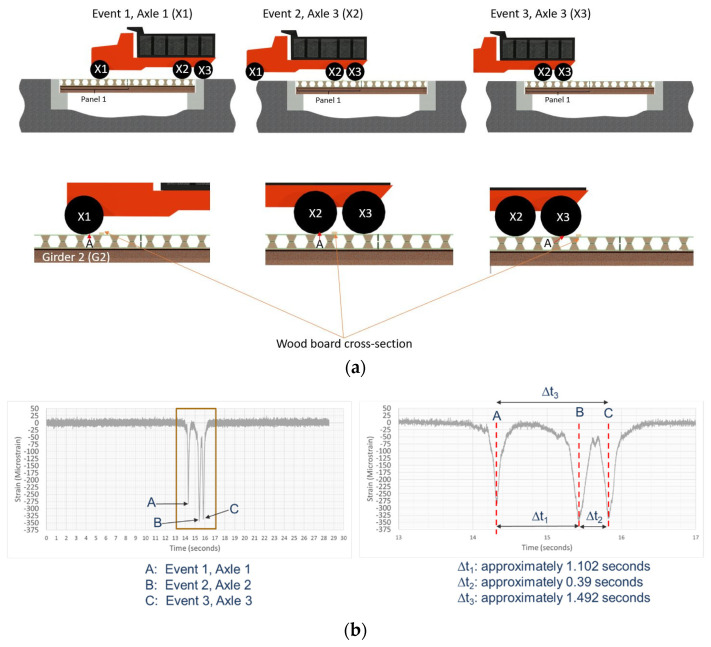
(**a**) Illustration of dynamic loading on composite deck panel for axle 1 (X1), axle 2 (X2), and axle 3 (X3), as all three axles contact the wooden board used to measure the FBG’s strain response. Point “A” denotes the datum reference for the sensor path layout for both HD-FOS and FBG on the panel deck. (**b**) Example of the corresponding strain response of FBG 2 to the three axles in contact with the wooden board when FRP deck Panel 1 is dynamically loaded.

**Figure 14:**
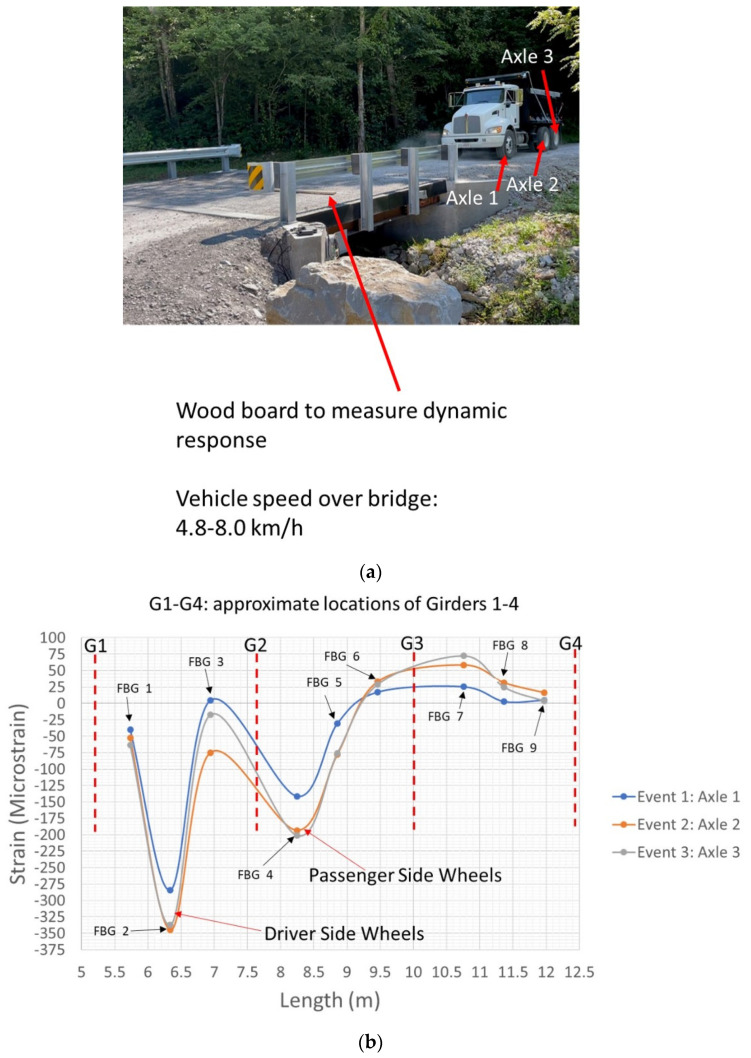
(**a**) Image of vehicle (dump truck) prior to traveling over the FRP deck panel bridge with a wooden board for dynamic load testing. (**b**) FBG’s strain responses to dynamic loading on FRP deck Panel 1 for axle 1 (X1), axle 2 (X2), and axle 3 (X2).

As shown in Figure 7b and Figure 13a, a wooden board (2.4 m length × 89 mm width × 38 mm thickness) was positioned on the bridge to amplify the detection of strain and vibration responses for dynamic loading. The FBG sensor was utilized to measure the strain response due to its advantages in a dynamic measurement scenario over the HD-FOS. Figure 13b and Figure 14, and Table 5 show examples of strain responses to dynamic loading on composite deck Panel 1 in three events (X1, X2, and X3) involving the axles’ contact with the wooden board. Three unambiguous compressive strain peaks are observed, corresponding to axle X1 (Event 1) at −284 με, increasing approximately to −345 με (21.5%); corresponding to axle X2 (Event 2) at −345 με, followed by a slight decrease (2.3%) to −337 με; and corresponding to axle X3 (Event 3). Obtaining the response of a bridge structure to moving vehicular loading for statically indeterminate structures is a very complex analytical problem, and the data measured using FBG sensors as shown in Figure 14 is very useful for developing new models for load distribution and bridge load rating. Additionally, the progressive increase in strain values along the length of the FBGs can be observed (as shown in Figure 14), with two compressive strain response peaks corresponding to FBG 2 (driver side) and FBG 4 (passenger side). FBG 4 (passenger side) had a lower compressive strain response compared with FBG 2, for example, a 36.9% increase in compression strain from −141 με to −193 με, followed by a slight increase of (4.1%) to −201 μm for FBG 4, corresponding to the passenger side. The FBG 2, located at approximately the 6.35 m position on the sensor as shown in Figure 14, is approximately located mid-span between girder G1 and girder G2. FBG 4 is located approximately at the 8.35 m sensor position, which is located approximately 0.608 m offset from the midspan point between girder G2 and G3. The FBG 2 exhibited higher compressive strain values due to the maximum moment (load × distance) induced by the passage of the vehicle over the midspan of the FRP deck panel between G1 and G2, compared to FBG 4 that experienced a lower moment due to the fact that FBG 4 was positioned closer to G2. FBG 3 unsurprisingly exhibited a smaller compressive behavior due to moment behavior between FBG 2 and FBG 4. A similar strain response was observed for the static response using HD-FOS (Figure 12) for the previously described difference in peak strain tensile and compressive values for the driver and passenger side. These results demonstrate the potential of utilizing both sensor types to effectively measure the static and dynamic responses of the FRP deck panel.

**Figure 15:**
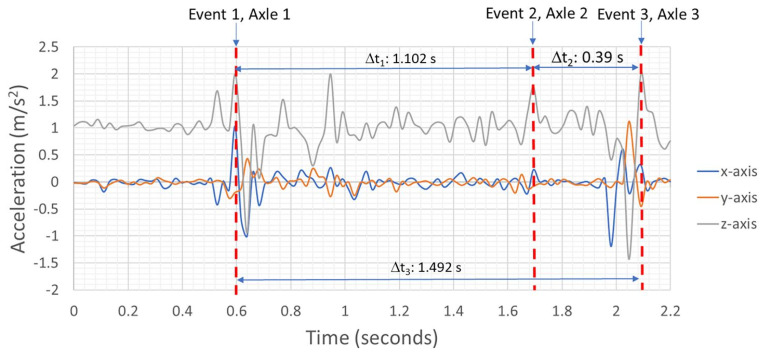
Accelerometer’s responses to vehicular dynamic loading on FRP deck Panel 1 in three events corresponding to the axles’ contact with the wooden board. Analysis in the frequency domain can reveal shifts in spectral content relatable to bridge health.

Figure 15 shows the accelerometer’s responses to the vehicle loading when the axles are in contact with the wooden board for the dynamic loading case study. The accelerometer’s responses in three directions (x, y, and z) are recorded above a threshold acceleration value to record events resulting from crashes, to sense any potential damage, and to alert inspection officials about the condition of the bridge through the cloud data interface. The triaxial accelerometer provides acceleration versus time for the given vehicular loading. Multiplying with the mass of the vehicle now precisely provides the force–time history. The FBG sensors provide the strain–time response. Thus, coupling the response of accelerometers with fiber optic sensors provides a comprehensive analysis of the bridge’s response to dynamic events. Since FBG sensors currently cannot be read in wireless mode, obtaining alerts from the bridge for a crash event on the bridge or guard rail system can only be possible utilizing a wireless sensing module.

**Figure 16:**
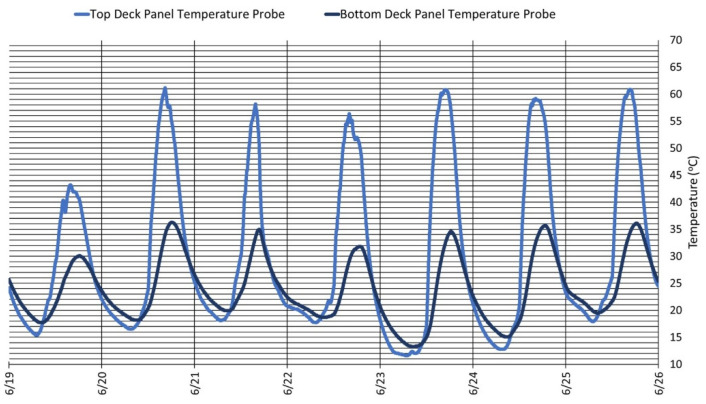
Structural temperature in the top and bottom panels of the FRP composite deck panel.

Figure 16 shows the temperatures observed by the top and bottom deck panel for the FRP deck panel over a 7 day period during the summer season in the month of June 2021. The digital thermometers embedded in the deck observed a mean peak temperature of approximately 59.9 ± 4.8 °C for the top deck panel and 34.1 ± 1.8 °C for the bottom deck panel. The mean peak temperature difference between the peak temperature for the top and bottom deck panels was 22.8 ± 3.0 °C. The digital thermometers embedded in the deck observed a mean trough temperature of approximately 15.8 ± 1.9 °C for the top deck panel and 17.7 ± 1.7 °C for the bottom deck panel. The mean trough temperature difference between the peak temperature for the top and bottom deck panels was 1.8 ± 0.2 °C. This is a novel aspect of a fiber-reinforced composite-material-based bridge deck, as we can integrate the digital thermometer sensors during manufacturing and embed them at different locations based on the targeted physical phenomenon being evaluated. By knowing the thermal conductivity and diffusivity, surface and other location temperatures through the thickness of the bridge deck can be obtained, if one were to use limited sensors in the through-thickness direction.

## 5. Conclusions

In this study, we have demonstrated the integration of fiber optic sensors during the manufacturing of a composite bridge deck for the first time. In addition, the coupling of high-density fiber optic sensors for exceptional spatial resolution (5 mm resolution over 20 m length) and integrating the high-speed response with Bragg gratings to monitor dynamic conditions is novel. We are also documenting for the first time comparative measurements of HDFOS with gratings-based fiber optic sensors. Since fiber optic sensing required monitoring with interrogators connected to sensors in the field on-demand, we also introduced the concept for the first time of having triggers to acquire and monitor fiber optic sensor data based on a remote wireless sensing module that provides environmental conditions and crash events. These three major concepts are highly novel and have been demonstrated for the first time in this paper. In this study, results associated with the use of glass fiber reinforced sandwich structures or use as extremely lightweight bridge decks are presented. The weight savings of 90% alone are substantial compared to traditional concrete bridge decks and facilitate the use of relatively simple lifting equipment for the installation of such fiber-reinforced bridge decks. The installation of the demonstration bridge required less than one day after the decks were manufactured for placement and alignment and joining with supporting steel girders. The joining of deck material with girders was undertaken in cold weather during the peak of winter to further demonstrate the viability of using this technology for regions with extreme weather conditions. A novel high-density fiber optic sensing technology was integrated with high-speed fiber Bragg grating sensors, and example results are demonstrated. Due to significant weight savings, the requirement for geotechnical design associated with foundation support also dramatically reduces along with associated gains in expected performance during earthquake-type loadings. A custom-developed wireless sensing package was integrated by the team to continuously monitor the bridge using high-resolution video, several temperature sensors measuring temperature through-thickness and spatially, and having the ability to measure deflection at target points of interest and use of a custom sensor to count the number of vehicles crossing the bridge. We have integrated two 20 m long fiber optic sensors with the ability to measure spatially resolved strain at exceptional resolution along thousands of points in the bridge deck.. Such technology can be easily integrated to existing concrete and steel bridges that are not in good condition to monitor their health and for revised load ratings and can be integrated on-demand, as well as using the cloud-computing-based wireless sensing options described in this paper.

The nature of this paper is to demonstrate the several novel concepts identified above and show example data. Engineers familiar with bridge structures will be conversant with how to use such data. For example, the strain measured from HDFOS provides spatially varying strain response to vehicular loading. Bridge structures are inherently statically indeterminate, and it is not easy to solve precisely the response of the bridge to external loading due to the difficulty in modeling realistically the support conditions (fixed, pin, roller-type supports) or nature of load partitioning. Thus, the strain response shown for static loading is useful for adding value to having proposed sensors integrated in the bridge deck. Similarly, if there was a crash event of an automobile with side guard rails, the FBG sensors can provide the dynamic response of the bridge structure, while accelerometer response can be used to obtain the dynamic force applied to the bridge structure. The HDFOS response after such an event is useful in evaluating the residual strength of the bridge deck.

## Figures and Tables

**Table 1:** A summary of the interrogator used for the evaluation of the FRP deck panel coupled with HD-FOS [54].

Manufacturer, Interrogator	Luna Innovations, ODiSI 6104
Measurement rate (Hz)	2.1
Gage pitch (mm)	2.6
Resolution (με)	0.1
Instrument accuracy (με)	±1
Measurement uncertainty (με)	±2
Strain measurement range (με)	±15,000

**Table 2:** A summary of the interrogator used for the evaluation of the FRP deck panel coupled with FBG [63].

Manufacturer, Interrogator	Luna Innovations, Hyperion i155
Scan rate (Hz)	1000
Wavelength range (nm)	1460–1620
Wavelength accuracy/stability (pm)	1
Dynamic range/continuous	25 dB peak/40 dB FS
Channel width (nm)	160

**Table 3:** A summary of the fiber Bragg sensors and the HD-FOS for the evaluation of the FRP deck panel [64,65].

Sensor	HD-FOS	FBG
Interrogator	ODiSi 6104	Hyperion si155
Approximate distance between sensor and interrogator(m)	52	10
Sensorized length (m)	20	15.6
Number of optical fibers	2	1
Number of independent measurement points	~16,000 *	9
Optical connectors	APC	APC
Reflection (%)	-	24.7–31.2

* Calculated using a spatial resolution of one measurement point every 2.6 mm along the optical fiber.

**Table 4:** Polynomial coefficient values to convert spectral shift (GHz) for the two HD-FOS sensors evaluating the FRP deck panel.

Strain Coefficients	*a* (με/GHz)	*b* (με/GHz^2^)
Top surface of deck Panel 1	−6.695107936859131	−6.594909791601822 × 10^−5^
Bottom surface of deck Panel 1	−6.698797225952148	−5.1488801545929164 × 10^−5^

**Table 5:** Strain response values corresponding to each axle’s dynamic loading on FRP deck Panel 1.

FBG ID	FBG 1	FBG 2	FBG 3	FBG 4	FBG 5	FBG 6	FBG 7	FBG 8	FBG 9
Event 1: Axle 1, X1 (microstrain)	−40	−284	5	−141	−31	17	25	3	5
Event 2: Axle 2, X2 (microstrain)	−53	−345	−75	−193	−78	33	58	31	16
Event 3: Axle 3, X3 (microstrain)	−64	−337	−17	−201	−76	29	73	25	4
FBG location along fiber sensor (m)	5.732	6.338	6.941	8.244	8.852	9.461	10.758	11.367	11.972

## Data Availability

Data is available upon reasonable request.
